# Alcohol, Syphilis, and Gonorrhea, on Death Certificates

**Published:** 1915-11

**Authors:** 


### The Department of Eugenics, Mental and Social Hygiene

CONDUCTED BY
DR. MALONE DUGGAN
813-814 Gibbs Building, San Antonio, Texas
All communications for this department should be sent to DR. DUGGAN

The following is an important suggestion pointed out in the *Bulletin of the American Social Hygiene Association:*

### Alcohol, Syphilis, and Gonorrhea, on Death Certificates.

A recent bulletin of the Department of Health of New York City says: "The Department of Health has long since felt the need of more accurate statistics of the influence of alcohol on mortality and has realized that numerous deaths wherein alcohol, if not the primary cause, at least played an important rôle, have been recorded without reference being made to this important etiological factor. This, of course, is true also of deaths in which gonorrhea or syphilis has played a part. We appreciate the numerous and weighty reasons that induce physicians, if not to hide, at least to withhold this information. On the other hand, we should be shirking our duty as health officers if we did not make serious attempts to secure this information in order that it may be made the scientific basis of a campaign against these grave menaces to public health.

"In order to secure some degree of correctness in the statistics bearing upon the influence of alcohol on the death rate, the Bureau of Records has long been directing confidential letters of inquiry to physicians who report deaths in which it is apparent from the cause specified that alcohol or venereal infection has been a factor in terminating life. This method, while it has been helpful, has not proven sufficiently effective, and the department, therefore, appeals to physicians through the *Bulletin* and asks that in every case where these factors have been a contributing cause of death, they state that fact upon the certificate. Any objection to this procedure may be met by the employment of medical terminology, such as Ethylism Neuritis (Ethylic), Ethylic Hepatitis, Salpingitis (probably Neisserian), Hepatitis (probably Spirochaetal).

"Still another plan is to send a brief note sealed with the certificate. This information thus obtained will be treated in the strictest confidence and the note immediately destroyed. We urge upon the physicians the broader duty which they owe the community not to allow their sense of obligation to patients to stand in the way of supplying the Department of Health with this most important information."

